# The molecular evolution of mammalian spermatogenesis

**DOI:** 10.1016/j.cdev.2023.203865

**Published:** 2023-09

**Authors:** Nils Trost, Noe Mbengue, Henrik Kaessmann

**Affiliations:** Center for Molecular Biology (ZMBH), DKFZ-ZMBH Alliance, Heidelberg University, Heidelberg, Germany

**Keywords:** Mammals, Testis, Spermatogenesis, Molecular evolution, Male germ cells, Spermatogonial stem cells

## Abstract

The testis is a key male reproductive organ that produces gametes through the process of spermatogenesis. Testis morphologies, sperm phenotypes, and the process of spermatogenesis evolve rapidly in mammals, presumably due to the evolutionary pressure on males to give rise to their own offspring. Here, we review studies illuminating the molecular evolution of the testis, in particular large-scale transcriptomic studies, which were based on bulk tissue samples and, more recently, individual cells. Together with various genomic and epigenomic data, these studies have unveiled the cellular source, molecular mechanisms, and evolutionary forces that underlie the rapid phenotypic evolution of the testis. They also revealed shared (ancestral) and species-specific spermatogenic gene expression programs. The insights and available data that have accumulated also provide a valuable resource for the investigation and treatment of male fertility disorders – a dramatically increasing problem in modern industrial societies.

## Highlights

• The testis is a rapidly evolving organ, on both the transcriptomic and translatomic layer.
• The force of sperm competition has shaped the evolution of this organ.
• Insights from new data provide a resource for future investigations of testicular molecular evolution and male fertility.

The testis is the male reproductive gonad in all bilaterian animals, including humans and other mammals. The function of the testis is to produce sperm, through the process of spermatogenesis, but also androgens, primarily testosterone. The testis has evolved rapidly in mammals, which is likely mainly due to positive selection associated with the force of “sperm competition”, which reflects the evolutionary pressure on males to be reproductively successful; that is, to fertilize female eggs and thus give rise to their own offspring (Birkhead and Pizzari, 2002; Ramm et al., 2014). This pressure has led to a rapid divergence of testis sizes, sperm production rates, sperm morphologies, and various other cellular traits between species during mammalian evolution, owing to major mating systems differences, especially with respect to the extent of female promiscuity (Birkhead and Pizzari, 2002; Ramm et al., 2014). Even closely related species, such as the great apes, have very different testis sizes and sperm/semen properties (Birkhead and Pizzari, 2002); for example, bonobos have the largest testicles among apes, likely reflecting the evolution of extreme levels of female promiscuous mating behavior, whereas gorillas, where opportunities for promiscuous mating in the silverback male harem are limited, have rather small testes.

Studies in the past two decades have revealed that the rapid evolution of the testis is reflected at the molecular level. In particular the advent of RNA sequencing (RNA-seq) protocols has facilitated global large-scale cross-species investigations. Here, we discuss the exciting fundamental insights that these novel approaches have provided into the molecular basis of testicular evolution and the peculiar features of spermatogenesis.

We set the stage by introducing the spermatogenic/testicular cell types and highlighting the peculiar chromatin dynamics of the autosomes and sex chromosomes during spermatogenesis. We then summarize how bulk tissue RNA-seq and ribosome profiling (or Ribo-seq) studies have shed light on the consequences of these chromatin dynamics but also on the extensive translational regulation that is occurring during spermatogenesis. We proceed by discussing insights into the molecular evolution of the testis obtained from cross-species comparisons of these data types, to then move on to describe how recent evolutionary single-cell RNA-seq studies have led to groundbreaking findings regarding the cellular source and evolutionary forces underlying the rapid evolution of the testis. Next, we delve into the intricate and intriguing patterns and mechanisms of sex chromosome evolution pertaining to spermatogenesis. We conclude our account by discussing overall implications of the recent large-scale genomics investigations of the testis and by identifying promising future research avenues.

## Spermatogenesis – cell types

**Fig. 1:**
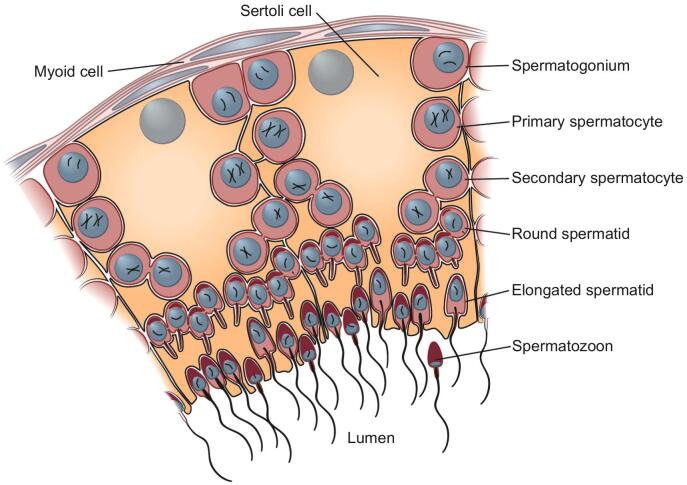
Major cell types in spermatogenesis. A segment of the cross section of a seminiferous tubule in the testes. In their development from spermatogonial stem cells to testicular spermatozoa, the cells move from the basal lamina of the seminiferous tubule towards the lumen, surrounded and nourished by Sertoli cells. The diploid spermatogonia differentiate into primary spermatocytes that undergo the first meiotic division, then called secondary spermatocytes, followed by the second meiotic division to form spermatids. These haploid cells differentiate from round spermatids to elongated spermatids and finally into immature spermatocytes, which detach from the Sertoli cells and travel to the epididymis, where they fully mature.

Spermatogenesis takes place in seminiferous tubules, which crowd the testis (Nishimura and L'Hernault, 2017). Specifically, spermatogenesis proceeds from the edge of the seminiferous tubules towards the lumen in the center. Spermatogenesis starts with cells termed spermatogonia. Spermatogonia, which derive from spermatogonial stem cells, fuel the process of spermatogenesis; that is, all other cell types arise from spermatogonia along a continuous cellular proliferation and differentiation path (Fig. 1). Notably, spermatogonial stem cells ultimately derive from primordial germ cells, which emerge early in embryonic development (e.g., week 4 of gestation in humans). Primordial germ cells differentiate into gonocytes during fetal development following sex determination, which then in turn differentiate into spermatogonial stem cells after birth.

During spermatogenesis spermatogonia differentiate into spermatocytes, where meiosis takes place (Fig. 1). The meiotic divisions lead to haploid spermatids, which are initially round and then elongate while concomitantly growing a flagellum. Elongated spermatids differentiate into spermatozoa, which end up in the lumen of the seminiferous tubules (Fig. 1). These immature spermatozoa then travel through the lumen to a testis-associated structure termed the epididymis, where they fully mature into spermatozoa with full motility and fertilization capabilities (Nishimura and L'Hernault, 2017). Notably, spermatogenesis is supported by large somatic Sertoli cells (Fig. 1). Other testicular cell types include Leydig cells, which are interstitial cells adjacent to seminiferous tubules that produce androgens, in particular testosterone, but also peritubular myoid cells (smooth muscle cells surrounding seminiferous tubules), endothelial (blood vessel) cells, and macrophages (an immune cell type).

## Extensive chromatin remodeling

**Fig. 2:**
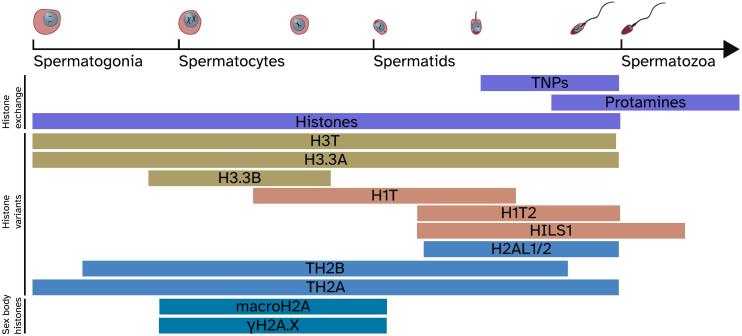
Testicular chromatin remodeling. Throughout spermatogenesis (black arrow) histones are exchanged by testis specific histone variants. In the postmeiotic spermatids histones are exchanged by transition proteins (TNPs), the TNPs in turn are exchanged by protamines. The protamines facilitate the dense packaging of chromatin in the sperm head. During meiosis the unsynapsed sex chromosomes become sequestered in the sex body and transcriptionally silenced. One hallmark feature of the sex chromosomes in the sex body is the presence of phosphorylated H2AX along the entire chromosome length. Data from Kimmins and Sassone-Corsi (2005), and Turner (2015).

Spermatogenesis displays several unique and peculiar molecular chromatin features. Chromatin in spermatogenic cells is indeed massively remodeled during spermatogenesis. This process involves repackaging of the DNA initially around testis-specific histones and histone variants, then around transition proteins, and in the end around small protamines, which allow dense packaging of the DNA into the compact sperm head (Kimmins and Sassone-Corsi, 2005) (Fig. 2). As discussed below, these chromatin dynamics are thought to have implications for transcription and, consequently, for molecular evolution. Interestingly, sex chromosomes are affected by an additional drastic chromatin remodeling event; namely, during meiotic sex chromosome inactivation (Turner, 2015) (MSCI), sex chromosomes are inactivated in pachytene spermatocytes following condensation into the so-called sex body, a process mediated by the accumulation of the phosphorylated form of histone H2AX (Fig. 2). MSCI also has profound implications for transcript abundances, messenger RNA translation and molecular evolution, as explained below. Notably, MSCI is a consequence of the fact that the highly differentiated X and Y chromosomes cannot synapse in pachytene spermatocytes and thus represents a sex chromosome-specific form of the general epigenetic phenomenon of meiotic silencing of unsynapsed chromatin (MSUC) (Turner, 2015).

## Global transcriptome and translatome dynamics

Molecular biologists have been aware for decades that the testis expresses many or even most genes – more genes than other organs – and have therefore, for example, often resorted to the testis for cloning experiments. Initial analyses of RNA-seq data derived from bulk tissue samples confirmed this notion at a genome-wide scale (Ramskold et al., 2009), indicating that the testis indeed displays an exceptional transcriptome complexity. RNA-seq-based investigations of the testis across mammals then revealed widespread transcription even in intergenic regions far away from genes, albeit at overall low levels (Soumillon et al., 2013). Notably, transcriptome analyses of cell populations enriched for individual spermatogenic cell types unveiled the cellular source of this widespread transcription: pachytene spermatocytes and especially round spermatids (Soumillon et al., 2013). Moreover and consistently, matched genome-wide epigenomic (i.e., histone and DNA methylation) data provided evidence for an overall loose (i.e., transcriptionally permissive) chromatin state in these cell types (Soumillon et al., 2013). Thus, the testis-specific histones and histone variants, introduced during the massive chromatin remodeling (see above; Fig. 2) in these cells, lead to an overall open and transcriptionally permissive chromatin state in spermatocytes and round spermatids, in agreement with the suggested properties of these histone types (Kimmins and Sassone-Corsi, 2005; Kleene, 2001). The repackaging process, which may require a loose packaging state to release/incorporate histones, may itself also contribute to an open chromatin state in these cells and thus further facilitate (low-level) transcription; that is, promiscuous binding of RNA polymerases. With respect to the sex chromosomes, studies based on bulk tissue/cell population RNA-seq data highlighted the massive reduction in transcript abundances during and after meiosis due to MSCI (Julien et al., 2012; Marin et al., 2017; Soumillon et al., 2013).

Recent genome-wide investigations of mRNA translation based on Ribo-seq (Ingolia et al., 2009) – a highly sensitive method that provides a good proxy for the rate of protein synthesis on the basis of deep sequencing of ribosome-protected mRNA fragments (‘ribosome footprints’) – revealed that translational regulation is particularly pronounced and intricate during spermatogenesis (Murat et al., 2023; Wang et al., 2020). Indeed, translation is overall rather repressed for many transcripts in spermatocytes and spermatids and/or partly delayed to later spermatogenic stages (Murat et al., 2023; Wang et al., 2020), consistent with early work in the field that was based on smaller scale molecular experiments (Kleene, 2001). A potential explanation for the delayed translation of transcripts is that the respective encoded proteins are only needed at very late spermatogenic stages (i.e., in elongated spermatids or spermatozoa), when the genome becomes transcriptionally inert due to global chromatin condensation following the wrapping of DNA around transition proteins and protamines (Kleene, 2001) (Fig. 2). An alternative, not mutually exclusive explanation is that translational repression in late spermatocytes and round spermatids may counteract the promiscuous transcription in these cell types due to the permissive chromatin state (see above) and thus prevent negative consequences of potentially deleterious proteins or protein fragments from open reading frames in intergenic regions (Kleene, 2001; Murat et al., 2023). Remarkably, analyses of these Ribo-seq datasets also revealed a strong increase of the efficiency of translation of transcripts derived from X chromosomal genes, which likely compensates for the strong reduction of transcript abundances due to MSCI and thus ensures adequate levels of X chromosome-derived proteins required during MSCI.

## Rapid molecular evolution of the testis

**Fig. 3:**
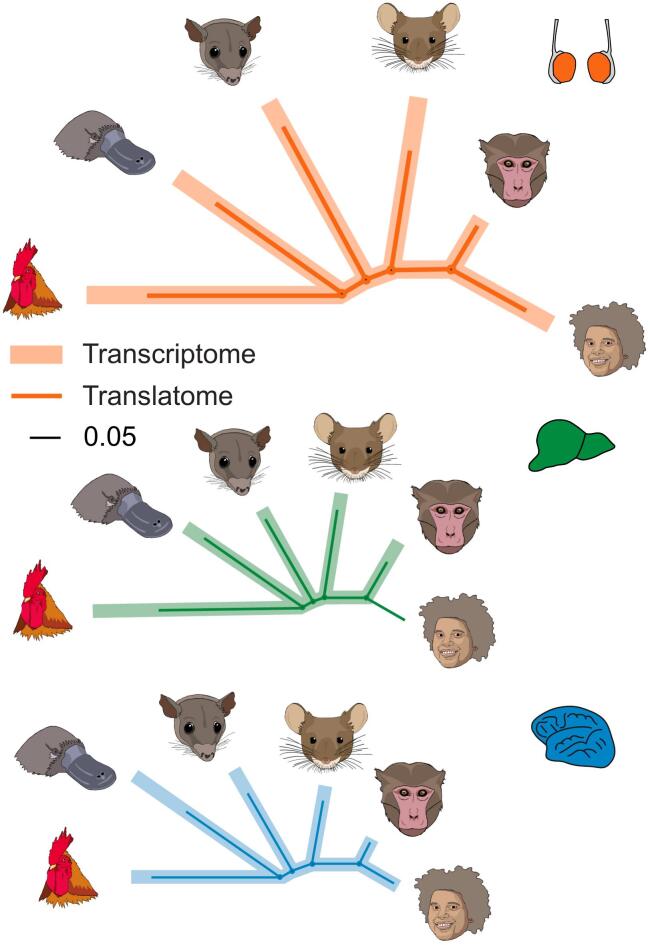
Evolution of gene expression. Phylogenetic analyses of the translatome (thin and dark) and transcriptome (thick and light) for testis, liver, and brain (from top to bottom) reveal the rapid evolution of the testis. The branch lengths indicate evolutionary changes in expression levels. Data from Wang et al. (2020).

Global comparisons of gene expression patterns across species based on bulk tissue samples provided initial insights into the rates of expression evolution in different mammalian organs. Early work, which was using microarrays to compare gene expression levels of corresponding one-to-one (1:1) orthologous genes between humans and chimpanzees for several organs, already showed that the testis had the greatest divergence in gene expression levels among the investigated organs (Khaitovich et al., 2005). Later studies, based on RNA-seq data, confirmed and extended this observation for both coding and noncoding genes across representative species that cover all major mammalian lineages (placental mammals, marsupials, and the egg-laying monotremes) (Brawand et al., 2011; Necsulea and Kaessmann, 2014; Necsulea et al., 2014). Thus, cross-mammalian gene expression trees – that is, trees based on differences in expression levels of 1:1 orthologous genes across mammals, showed that the overall branch lengths are longer for trees built for the testis than for other organs (Fig. 3), which implies that gene expression levels have changed more in the testis during evolution. Altogether, these bulk-tissue studies suggested that the rapid evolution of the testis at the phenotypic level (Birkhead and Pizzari, 2002; Ramm et al., 2014) is reflected at the molecular level (Khaitovich et al., 2006; Necsulea and Kaessmann, 2014). It was suggested that the accelerated gene expression evolution in the testis is due to positive selection associated with the force of sperm competition but may partly also reflect a reduction in purifying selection (Khaitovich et al., 2006; Necsulea and Kaessmann, 2014; Wong, 2010) (see below for a detailed discussion of the forces and mechanisms underlying the rapid molecular evolution of the testis). It is noteworthy in this context that the brain stands out at the other of the evolutionary rate spectrum, with a particularly low rate of transcriptome evolution (Khaitovich et al., 2006; Necsulea and Kaessmann, 2014). This observation may seem remarkable in view of the substantial changes in the size, structure, and cellular composition of the brain that occurred during mammalian evolution, but is consistent with previous findings (e.g., the low rate of coding sequence change of brain-expressed genes), which suggested that nervous tissues may have more fine-tuned expression networks than other organs (Khaitovich et al., 2006; Necsulea and Kaessmann, 2014).

Notably, our recent work has brought the investigation of gene expression evolution from the transcriptome to the next expression layer – the translatome – based on Ribo-seq data for several organs (including the testis) from representative mammals (Wang et al., 2020). This study showed that the rapid evolution of the testis compared to that of other organs also manifests itself at this second major expression layer (Fig. 3). Interestingly, however, the overall rate of expression evolution is lower across organs at the translatome than at the transcriptome layer (Fig. 3), given that evolutionary changes in transcript abundances are often offset by counteracting translational changes in the opposite direction (Wang et al., 2020).

## Evolution of spermatogenesis at the cellular level

**Fig. 4:**
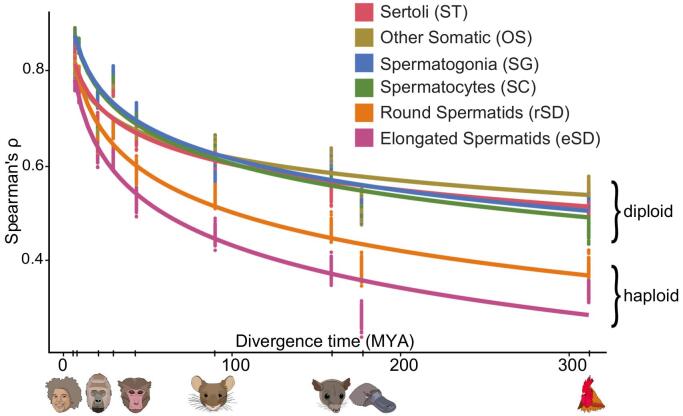
Different rates of evolution in testicular cell types. Pairwise Spearman's rank correlation coefficient between human and other species for testicular cell types. The haploid cell types diverge faster than the diploid cell types. Adapted from Murat et al., Nature 2022, https://doi.org/10.1038/s41586-022-05547-7 | CC BY 4.0.

Recent high-throughput single-cell (sc) or single-nucleus (sn) RNA-seq technologies have enabled detailed investigations of the cellular and molecular evolution of organs. We thus recently performed an extensive evolutionary single-cell transcriptome study for testes from eleven species that cover the three major mammalian lineages (placental mammals, marsupials, monotremes) and birds (the evolutionary outgroup), and include seven primates (Murat et al., 2023) (Fig. 4). Notably, this work also represented the first cross-mammalian single-cell transcriptome investigation of an organ in general.

To trace the cellular source of the rapid evolution of the testis, we built expression trees for the different cell types based on these data and found that the total branch lengths of the trees, which reflect the amount of evolutionary expression change (akin to the aforementioned bulk RNA-seq work), vary substantially between cell types. Interestingly, while the rate of expression evolution is similar in Sertoli cells and diploid spermatogenic cells (and lower than that in other somatic cell types), it was found to be substantially higher in the post-meiotic haploid cell types (i.e., round and elongated spermatids), consistent with previous work and a recent inference based on data for three placental mammals (Good and Nachman, 2005; Shami et al., 2020). Pairwise species comparisons confirmed the rapid expression evolution of post-meiotic cell types across amniotes (i.e., mammals and the bird) (Fig. 4). Altogether, these results therefore revealed that the rapid evolution of the testis is primarily driven by changes in late spermatogenic cell types.

## Evolutionary forces underlying rapid evolution of late spermatogenesis

We sought to trace the evolutionary forces underlying the rapid evolution of late spermatogenesis and reasoned that two non-mutually exclusive patterns of natural selection may account for this finding (Murat et al., 2023). First, later stages of spermatogenesis might evolve under weaker purifying selection (i.e., reduced functional constraints) and hence facilitate evolutionary change. Second, the greater divergence in later stages might at least partly be driven by stronger positive selection, increasing the rate of fixation of adaptive evolutionary innovations. Detailed analyses that incorporated other datasets uncovered that indeed both patterns of selection contribute to the rapid evolution of late spermatogenesis.

Specifically, signatures of functional constraints along spermatogenesis were assessed based on patterns of the tolerance to functional mutations, mouse knockouts, and rates of amino acid altering substitutions in coding sequences of genes expressed in different spermatogenic stages. All of these analyses showed a progressive reduction of constraint towards late stages of spermatogenesis across species, typically with the strongest reduction in early spermiogenesis; that is, in round spermatids (Murat et al., 2023).

**Fig. 5:**
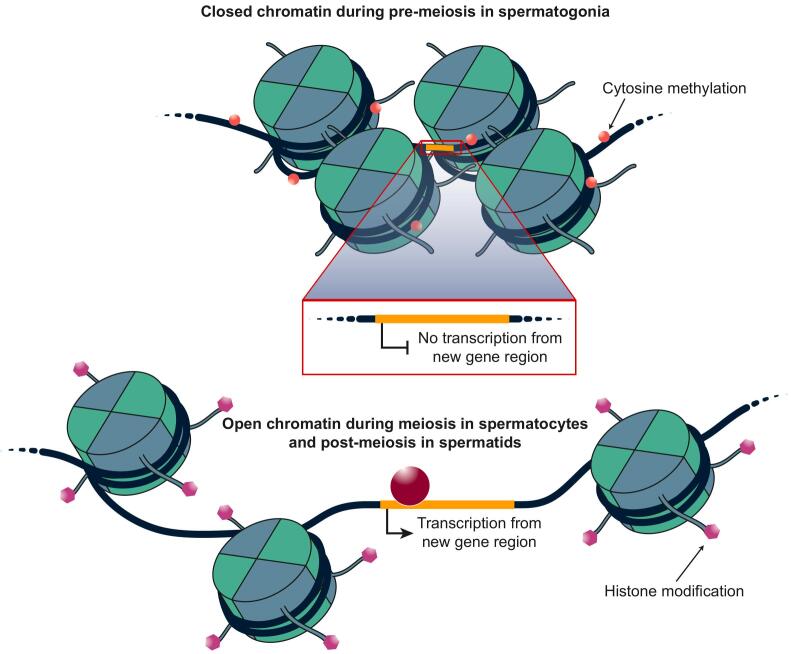
Chromatin confirmation changes during spermatogenesis. The chromatin conformation in spermatogenesis changes from closed (compact) in pre-meiotic spermatagonia to open (relaxed) in meiotic spermatocytes and post-meiotic spermatids. The extensive remodeling is facilitated by histone modifications (acetylation and methylation) and widespread demethylation of dinucleotide-enriched promoter sequences. This permissive chromatin environment allows for the initial transcription of new genes during evolution.

On the other hand, an examination of the temporal expression pattern of genes whose encoded protein sequences have been shaped by positive selection revealed a significant increase in percentages of positively selected genes used during spermatogenesis, with a peak in round spermatids (Murat et al., 2023). Moreover, given that new genes that emerged during evolution also contribute to evolutionary innovations, we scrutinized the temporal contribution of recently emerged genes to gene expression programs in germ cells and found that transcriptomes indeed become younger during spermatogenesis. This indicated that new genes have increasingly more prominent roles in later stages, particularly in round spermatids, consistent with previous observations (Lau et al., 2020; Shami et al., 2020). As discussed above, previous work based on bulk cell type analyses in mouse (Soumillon et al., 2013) uncovered a transcriptionally permissive chromatin environment during spermatogenesis, in particular in round spermatids (Fig. 5). Notably, this permissive chromatin environment is thought to have facilitated the emergence of new genes during evolution – the “out of the testis” scenario for the birth of new genes, which posits that new genes initially obtained functions in the testis and then – later in evolution and following regulatory mutations leading to more widespread expression – in other organs (Kaessmann, 2010; Necsulea and Kaessmann, 2014; Soumillon et al., 2013). In agreement with this notion, our analyses detect in all species significantly increased contributions of intergenic transcripts after meiosis and a parallel decrease in the contributions of protein-coding genes.

But what are the fundaments underlying the dynamic changes of selective forces and patterns of innovation during spermatogenesis? Given that the breadth of expression across tissues and developmental processes (termed “expression pleiotropy”) was proposed to represent a main determinant of the types of mutations that are permissible under selection (Carroll, 2005), we assessed patterns of expression pleiotropy across spermatogenesis using our spatiotemporal transcriptome data for several mammalian organs (Cardoso-Moreira et al., 2019). This analysis revealed that genes used later in spermatogenesis, in particular those in round spermatids, have substantially more specific spatiotemporal profiles than genes employed earlier in spermatogenesis and in somatic cells. Because a decrease in expression pleiotropy can explain both a decrease in functional constraints and an increase in adaptation (Cardoso-Moreira et al., 2019; Carroll, 2005), we suggested that it likely is a major contributor to the accelerated molecular evolution in late spermatogenesis (Murat et al., 2023). Additionally, the specific type of selection acting on haploid cells (Immler, 2019) (haploid selection), where expressed alleles (even recessive ones) are directly exposed to selection, might have contributed to the exceptionally rapid evolution of round spermatids. We suggest that all of the aforementioned mechanisms and selective forces facilitated the rapid evolution of late spermatogenesis, which was ultimately driven by the force of sperm competition.

## Conserved and diverged gene expression programs

**Fig. 6:**
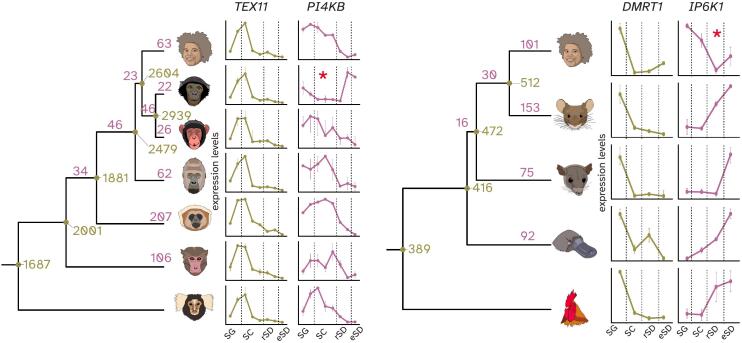
Gene expression program evolution. Analysis of gene expression trajectories along spermatogenesis in primates (left) and amniotes (right) identified both gene expression trajectories conserved across branches (in olive) and lineage specific trajectory changes (in purple). *PI4KB* and *IP6K1* are examples for changed expression trajectories with the asterisk denoting on which branch the change was called. *TEX11* and *DMRT1* are examples for conserved expression trajectories. Adapted from Murat et al., Nature 2022, https://doi.org/10.1038/s41586-022-05547-7 | CC BY 4.0. (For interpretation of the references to colour in this figure legend, the reader is referred to the web version of this article.)

In our study (Murat et al., 2023), we also traced individual genes underlying conserved (ancestral) and diverged aspects of germ cells by comparing expression trajectories along spermatogenesis of 1:1 orthologous genes across species (Cardoso-Moreira et al., 2019). These analyses identified thousands of genes with conserved expression trajectories across different lineages or species (Murat et al., 2023) (Fig. 6), which likely reflect core ancestral gene expression programs of different groups of mammals. By contrast, we also detected hundreds of genes with evolutionary trajectory changes (Murat et al., 2023) (Fig. 6), which probably contributed to phenotypic innovations during evolution.

Notably, integrated analyses of our data and known fertility phenotypes (Koscielny et al., 2014) unveiled that genes involved in fertility are significantly more conserved in their expression trajectories than genes not associated with fertility (Murat et al., 2023). This finding implies that genes with conserved expression trajectories for which spermatogenesis functions have remained uncharacterized represent promising candidates for the exploration of fertility phenotypes in the future.

## Sex chromosomes

Finally, we used our single-nucleus spermatogenic data to scrutinize expression patterns of sex chromosomal genes and their evolution across mammals (Murat et al., 2023). Two sets of sex chromosomes emerged in mammals from different pairs of ancestral autosomes around 180 million years ago (Cortez et al., 2014). The therian XY chromosome system originated just prior to the split of placental mammals and marsupials and therefore has largely evolved independently in these two lineages. Roughly at the same time, the monotreme sex chromosome system arose from a different pair of autosomes and eventually expanded to a striking number of five pairs of XY chromosomes (Cortez et al., 2014). The formation of these sex chromosomes involved substantial changes of gene repertoires and expression patterns due to structural changes and sex-related forces of natural selection (Necsulea and Kaessmann, 2014).

**Fig. 7:**
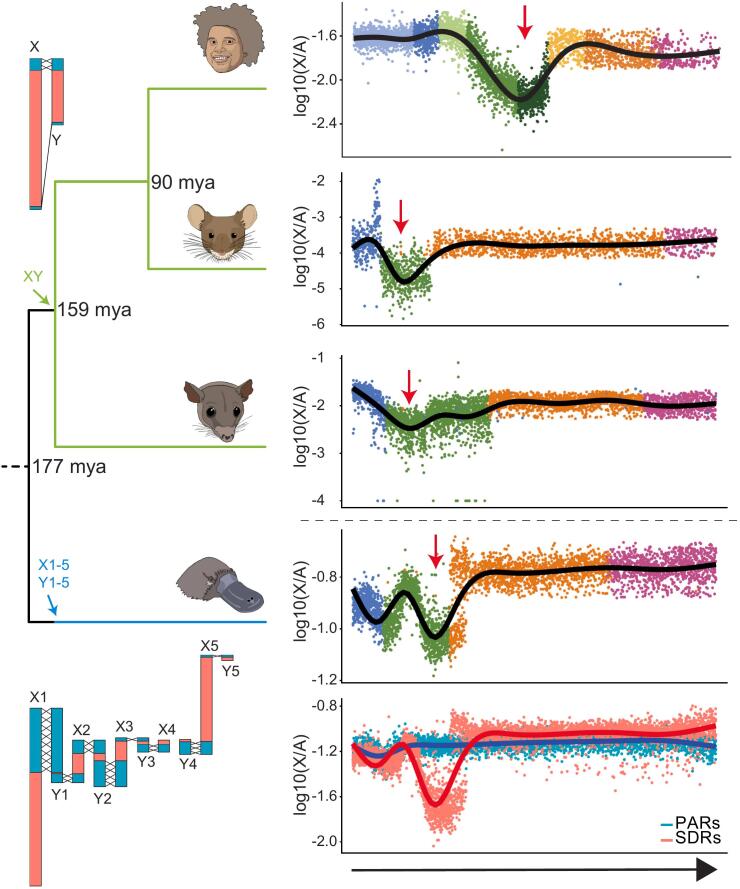
Sex chromosome dynamics during spermatogenesis. The mammalian sex chromosome systems originated after the split of therians (placental mammals and marsupials) and monotremes. The therian sex chromosomes in the XY chromosome system contain large sexually differentiated regions (SDRs), which are not homologous between the X and Y, preventing synapsis during meiosis. The small pseudoautosomal regions (PARs), which are homologous between the X and the Y can synapse during meiosis. The 5 X and 5 Y chromosomes of monotremes in contrast contain large PARs and only small SDRs (left). During spermatogenesis (indicated by arrow) the sex chromosomes become inactivated in pachytene spermatocytes. In platypus this inactivation can only be observed in the unsynapsed SDRs. Adapted from Murat et al., Nature 2022, https://doi.org/10.1038/s41586-022-05547-7 | CC BY 4.0.

Our analyses provided several intriguing insights into the expression of sex chromosome-linked genes and their evolution (Murat et al., 2023). First, we uncovered that genes with predominant expression in spermatogonia and Sertoli cells have accumulated on the X chromosome during mammalian evolution in both sex chromosome systems ever since their emergence. We hypothesized that this enrichment was driven by the unique selective environment on this chromosome. That is, male-beneficial mutations, including those that are recessive, are always exposed to selection on the X chromosome, owing to its single-copy (hemizygous) status in males (Necsulea and Kaessmann, 2014; Rice, 1984; Wang et al., 2001). Second, we used our unique snRNA-seq-based data to separate X-and Y-bearing spermatids, with the aim to scrutinize their specific transcriptomal characteristics during spermiogenesis. Such a separation was previously not possible based on scRNA-seq data, given that X and Y spermatids remain connected by cytoplasmic bridges and therefore are thought to contain largely similar cytoplasmic transcript pools (Braun et al., 1989). We used our data to uncover global transcriptomal differences between the two types of spermatids, which uncovered, as expected, mostly differentially expressed sex-chromosomal genes, but, interestingly, we also identified autosomal genes with distinct transcript abundances, which might reflect trans-regulatory effects involving the sex chromosomes. Third, we then used these data to examine gene expression across spermatogenesis separately for X- and Y-linked genes, which revealed a substantial dip in sex chromosome transcript abundances around the pachytene stage of meiosis across both therian (i.e., placental mammal and marsupial) sex chromosome systems (Fig. 7), reflecting the process of MSCI, consistent with previous work (Turner, 2015). Evidence from previous work suggested that MSCI is absent in monotremes, implying that MSCI originated in the therian ancestor after the separation from the monotreme lineage (Daish et al., 2015). However, our analyses, which profited from a new high-quality platypus genome version (Zhou et al., 2021), showed a clear and strong signal of MSCI during platypus spermatogenesis, and that MSCI is as efficient in monotremes as it is in therians. Thus, our work unveiled that the mechanism of MSUC, which underlies MSCI (Turner, 2015), is an ancestral mammalian feature.

## Outlook

Here we have reviewed cross-mammalian transcriptome studies that – initially at the bulk-tissue level and more recently at the level of individual cells – have unveiled details of the unique and complex biology and molecular evolution of the testis and the process of spermatogenesis that takes place in this organ. The available data also provide a rich resource not only for future investigations of the molecular evolution across mammals but also for studies of the biology of the testis and associated fertility disorders – a dramatically increasing problem especially in industrialized regions of the world (Skakkebaek et al., 2022). In the future, analyses of new complementary datasets will complement the available data and insights. We foresee various interesting research endeavors. For example, scRNA-seq data for representative mammals will be valuable for inferring transcriptome patterns unique to the cytoplasm and single-cell full-length transcript data will allow assessments of the pronounced isoform diversity of the testis (Soumillon et al., 2013) and its evolution. To more directly assess the link between the extent of sperm competition and the rate of expression change during spermatogenesis and test previous hypotheses (Dapper and Wade, 2016; Wong, 2010), it will be interesting in the future to contrast lineages where strong sperm competition (e.g., chimpanzee and bonobo) with those where it is expected to be weak (e.g., gorilla). This will require extensive additional data, which may provide enough statistical power for this work (so far, such analyses have been limited by the short divergence times of these ape lineages). Single-cell translatome data (VanInsberghe et al., 2021) will be key to understand the contribution of post-transcriptional changes (Wang et al., 2020) to the evolution of spermatogenesis. Finally, cross-species and state-of-the-art epigenomic/gene regulatory data, such as snATAC-seq, will enable intriguing investigations of the regulatory basis of the gene expression programs underlying spermatogenesis and their evolution; this work will be especially interesting in view of the elaborate chromatin remodeling during the formation of male germ cells. Altogether, future work is thus bound to uncover further fascinating facets of the molecular evolution of the testis and shed further light on the molecular basis of infertility disorders.

## CRediT authorship contribution statement

Nils Trost designed and contributed figures and co-wrote the manuscript.

Noe Mbengue designed and contributed figures and co-wrote the manuscript.

Henrik Kaessmann wrote the manuscript.

## Funding sources

This review and the new research in our group covered at the end of this review by Murat et al. were supported by grants from the German Research Council (DFG, SFB 873) and the 10.13039/501100000781European Research Council (615253, OntoTransEvol) to H.K.
